# Immunoendocrine Interactions during HIV-TB Coinfection: Implications for the Design of New Adjuvant Therapies

**DOI:** 10.1155/2015/461093

**Published:** 2015-05-14

**Authors:** Guadalupe Veronica Suarez, Maria Belen Vecchione, Matias Tomas Angerami, Omar Sued, Andrea Claudia Bruttomesso, Oscar Adelmo Bottasso, Maria Florencia Quiroga

**Affiliations:** ^1^Instituto de Investigaciones Biomédicas en Retrovirus y SIDA, University of Buenos Aires School of Medicine, C1121ABG Buenos Aires, Argentina; ^2^Unidad de Microanálisis y Métodos Físicos Aplicados a Química Orgánica (UMYMFOR), Department of Organic Chemistry, University of Buenos Aires School of Sciences, C1428EGA Buenos Aires, Argentina; ^3^J.A. Fernández Hospital, Infectious Diseases Unit, C1425AGP Buenos Aires, Argentina; ^4^Huesped Foundation, C1202ABB Buenos Aires, Argentina; ^5^Instituto de Inmunología Clínica y Experimental de Rosario (IDICER), CONICET-UNR, Rosario, S2002LRL Santa Fe, Argentina

## Abstract

Worldwide, around 14 million individuals are coinfected with both tuberculosis (TB) and human immunodeficiency virus (HIV). In coinfected individuals, both pathogens weaken immunological system synergistically through mechanisms that are not fully understood. During both HIV and TB infections, there is a chronic state of inflammation associated to dramatic changes in immune cytokine and endocrine hormone levels. Despite this, the relevance of immunoendocrine interaction on both the orchestration of an effective immune response against both pathogens and the control of the chronic inflammation induced during HIV, TB, or both infections is still controversial. The present study reviews immunoendocrine interactions occurring during HIV and TB infections. We also expose our own findings on immunoendocrine cross talk in HIV-TB coinfection. Finally, we evaluate the use of adrenal hormones and their derivatives in immune-therapy and discuss the use of some of these compounds like the adjuvant for the prevention and treatment of TB in HIV patients.

## 1. Introduction

Worldwide, around 14 million individuals are believed to be coinfected with both tuberculosis (TB) and human immunodeficiency virus-1 (HIV-1), constituting together the leading infectious diseases in resource-limited countries [1].

The African region accounted for most of the HIV-infected TB cases, and TB is the first cause of death in AIDS context [1, 2]. Also, HIV coinfection raises latent TB reactivation risk by 20-fold, becoming the leading risk factor for* Mycobacterium tuberculosis* (*Mtb*) infection progression to active disease [1, 3]. Likewise, TB exacerbates HIV infection progression [4, 5]. Therefore, the intersecting HIV and tuberculosis epidemics in countries with a high disease burden of both infections pose many challenges and opportunities.

In coinfected individuals,* Mtb* and HIV, weaken immunological system synergistically, disrupting immune functions through mechanisms that are not fully understood.

An extended TB treatment (6 months) is required to eradicate* Mtb* infection, rising up to 9 months for HIV patients [6]. While replicating bacilli are killed by anti-TB drugs during the first weeks of treatment,* Mtb* evades drugs and host-immune responses by successfully adapting to a quiescent physiological state [7]. In the absence of chemotherapy treatment, immune system failure, among other factors, leads to TB relapse [8]. Therefore, a more effective and shorter treatment may require the modulation of the immune response towards a protective phenotype. Actually, several immune modulation strategies, like vitamin D supplement [9], treatment with Th1 cytokines [10, 11], and immune suppression to disrupt granulomas and make bacteria more susceptible to chemotherapy [12], among others, have been explored with diverse results [13].

Endocrine and immune systems are strictly connected by multiple mutual regulatory pathways, the most extensively studied of which is the anti-inflammatory and immune-suppressive action of glucocorticoids (GCs). Several cytokines have been found to regulate the endocrine system and many steroid and nonsteroid hormones have shown immunomodulatory effects [14–17].

In a broad range of bacterial, viral, and parasitic infections, the immune response developed against the pathogen is paralleled by a significantly altered hormonal response both in experimental models and human patients [18]. In fact, there is some evidence that supports a role for the interaction between immune and endocrine systems in the orchestration of an effective defense against the infectious agents [18, 19]. Despite this, the relevance of the cross talk between immune and endocrine systems on the defense against infections has not been fully elucidated.

The classical view stands that proinflammatory cytokines (mostly IL-1*β*, IL-6, TNF-*α*, and IFN-*α*/*β*) produced in response to pathogens induce the hypothalamic-pituitary-adrenal (HPA) axis for the release of GCs [14, 18]. In turn, GCs suppress the immune system at several levels, avoiding the possible adverse effects of an exacerbated immune response and helping to terminate it once the injurious agent was cleared [20]. However, although true for some particular situations like acute inflammation, the view of GCs such as simple immune suppressors has been changed, as there is growing evidence showing that GCs may be both pro- and anti-inflammatory, and also even necessary for several immune processes [20].

On the other hand, the activation of HPA axis induces the release of other adrenal hormones, like dehydroepiandrosterone (DHEA) and DHEA-sulphate (DHEA-s), with known anti-GC functions [21]. GCs and DHEA have opposite effects on adaptive immune cells. While GCs inhibit both Th1 and Th2 cytokines by activated human T cells, its effect on Th1 cytokines is more pronounced, shifting T cell response towards Th2 profiles [22]. On the contrary, DHEA promotes a shift towards Th1 responses by skewing cytokine production, upregulates Th1 cytokines like IL-2 and IFN-*γ*, and downregulates Th2 cytokines like IL-4 and IL-5 [23]. In line with this, DHEA and its derivatives have been proved to enhance protective immune responses against several pathogens [24–27].

During infection, immune-endocrine interactions are even more complex, since pathogens* per se* can modulate endocrine function either by releasing soluble factors or by directly colonizing endocrine tissue [28, 29]. Furthermore, specific treatments like antibiotics may affect hormone metabolism [30]. Moreover, during chronic infections like HIV or TB, in which pathogens are not cleared and immune response must be sustained in time, there is a chronic state of inflammation associated to dramatic changes in immune cytokine and endocrine hormone levels [29, 31]. Despite this, the relevance of immunoendocrine interaction on both the orchestration of an effective immune response against both pathogens and the control of the chronic inflammation is still controversial.

The present study reviews immunoendocrine interactions occurring during HIV and TB infections. In addition, we expose our own findings on immunoendocrine cross talk in HIV-TB coinfection. Finally, we evaluate the use of adrenal hormones and their derivatives in immune-therapy and discuss the use of some of these compounds like the adjuvant for the prevention and treatment of TB in HIV patients.

## 2. Immunoendocrine Alterations during Human Tuberculosis

Initial infection with* Mtb* occurs upon inhalation of airborne droplets containing bacilli, which, once in the lungs, infect and reside mainly in myeloid cells, especially alveolar macrophages [7]. In most people, such initial infection resolves, or it is kept under control by the development of an appropriate adaptive immune response that prevents bacillary proliferation and dissemination.

Effective control of* Mtb* infection relies on cell-mediated immunity [32]. Once in the infected tissue, T cells produce interferon gamma (IFN-*γ*) in response to mycobacterial antigen presentation by antigen presenting cells and consequently IFN-*γ* activates macrophages to kill intracellular bacteria [33, 34]. In addition to CD4+ T cells, which are thought to be the most important, both CD8+ T cells [35, 36] and CD1-restricted unconventional T cells [37] contribute to the effective control of* Mtb* infection. Both CD8+ T cells and CD1-restricted unconventional T cells are thought to be particularly important in the prevention of latent TB reactivation [35–38].

Moreover, at least 30% of the persons that take contact with* Mtb* remain persistently infected without exhibiting signs of disease in a state known as latent TB infection (LTBi) [39]. While infection may persist for life, bacillary growth may be reactivated in 5–10% of non HIV-infected subjects, probably due to factors affecting the host's immune status, and postprimary disease ensues [7]. As described above, in HIV-infected persons this rate rises to 20%. Persistent infection with* Mtb* is accompanied by chronic, low-level inflammation, which together drive to several changes in cytokine and hormone levels.

TB patients show a twofold increase in IFN-*γ* and IL-10 and a 10-fold increase in IL-6 plasma levels, accompanied by a 50% decrease in testosterone and DHEA in plasma when compared to healthy controls [40]. Also, TB patients show higher diurnal peaks of cortisol [41] and higher cortisol responses to ACTH stimulation [42], showing that HPA axis is activated in these patients. Since GCs enhance Th2 activity, HPA axis control may be relevant to the Th1/Th2 balance during the establishment of the anti-infectious immune response [33, 34, 43]. Conversely, DHEA can directly and indirectly enhance Th1 T cell activity [21]. According to this,* Mtb*-specific IFN-*γ* secretion in TB patients correlated positively with DHEA plasma levels and inversely with cortisol/DHEA ratio [44].

In addition to proinflammatory cytokines, regulatory cytokines such as TGF-*β* are likely involved in the control of some endocrine functions during TB infection. For instance,* in vitro* treatment of the human adrenal cell line NCI-H295-R with culture supernatants of* Mtb*-stimulated PBMC from TB patients inhibited DHEA and induced cortisol secretion [40]. Of note, treatment with anti-TGF-*β* antibodies abolished the inhibitory effect on DHEA but not cortisol secretion [45].

Overall, the hormonal changes occurring during* Mtb* infection could prevent the development of protective immune responses and the control of persistent inflammation.

## 3. Immunoendocrine Interactions in HIV Infection

HIV-1 infection is accompanied by a strong innate and adaptive immune response, beside which it remains as a chronic infection through lifetime.

The main obstacle for HIV eradication is the establishment of a latent HIV reservoir, which occurs during the very early stages of primary infection. Virus genomic integration and latency, as well as an enormous genetic diversity, propel a constant immune escape which limits HIV-specific immune response efficacy, especially during the early events of infection.

HIV infection causes the depletion of CD4+ T cells, which accounts for most infection hallmark symptoms. Additionally, a systemic and chronic immune activation and an accelerated T cell turnover are also thought to contribute to HIV disease progression [46]. Finally, the constant antigenic stimulation is thought to induce T cell dysfunction in response to antigen stimulation [47].

Despite the persistent immune escape seen in the great majority of HIV-infected people, there are rare individuals who control HIV-1 burden to undetectable levels. These individuals, known as elite controllers, show HIV-specific CD8+ T cells with higher cytotoxic capacity [48, 49] and are also associated to some specific MHC class I alleles [50]. In line with this, HIV infection progression was suggested to be associated with a reduction in cellular immunity, that is, Th1, and an increase in Th2 cytokine production [51, 52].

Considering that GCs provoke a shift from Th1 to Th2 immunity and also alter the balance between Th17 and Treg cells [53], recently implicated in the pathogenesis of HIV [54], HPA axis activation could play a central role in the progression of HIV infection to disease. In fact, increased basal ACTH and/or cortisol levels in 50% of HIV-infected patients accompanied by an impaired ACTH and cortisol response to stress and CRH challenge, especially in advanced disease, have been reported [55, 56]. The proposed mechanisms to this hypercortisolemia include a shift in steroidogenesis from DHEA and aldosterone to cortisol and the stimulation of hypothalamus, pituitary, and adrenal cortex by cytokines [56, 57]. Moreover, the stimulation of the HPA axis may also result from the stimulatory effects of viral proteins, like the HIV envelope protein gp-120 and the structural protein viral protein R (Vpr) which have been shown to increase serum ACTH and to cause GCs hypersensitivity, respectively [58–60].

HIV-infected patients show a characteristic pattern of high cortisol and low DHEA-s levels [61], which have been associated with a negative course of disease [55, 61]. In fact, it has been shown that DHEA-s levels decline with the progression of the disease in parallel with the CD4 T cell count [61]. Immune system deterioration in HIV-infected patients with high cortisol/DHEA-s levels seems to be associated with the suppression of Th1 cytokines and the concomitant increase in Th2 cytokines levels [62]. In line with this, it has been suggested that cortisol-resistant HIV-infected patients have a prominent Th1 cytokine profile and, therefore, present a limited progression to AIDS [63, 64].

## 4. Immunoendocrine Alterations Observed in HIV-TB Coinfection

There is limited data available about the role of adrenal steroids on the immune response developed by patients coinfected with HIV-1 and* Mtb*. We determined DHEA, DHEA-s, and cortisol plasma levels and the role of these adrenal hormones on* Mtb*-specific Th1 responses and Treg frequencies in patients dually infected with* Mtb* and HIV undergoing different stages of* Mtb* infection [65]. We observed that, in HIV-infected patients with active TB (HIV-TB), DHEA plasma levels were diminished by twofold compared to HIV-infected patients without* Mtb* coinfection or healthy donors. On the contrary, HIV-infected patients latently infected with* Mtb* (HIV-LTBi) showed preserved DHEA levels. Additionally, while cortisol plasma levels were slightly higher in HIV-TB patients than in HIV patients, cortisol/DHEA ratio was almost 4 times higher in HIV-TB patients compared to HIV, HIV-LTBi, and healthy donors. Of note, in HIV-TB patients, DHEA-s levels correlated positively, while cortisol plasma levels correlated negatively with CD4+ T cell count. Moreover, we observed an inverse correlation between DHEA-s plasma levels and Treg frequency in the same group.

A remarkable finding of this study was the persistent observation of a conspicuous CD4+CD25−FoxP3+ population in HIV-TB patients that was not observed in the other groups. Notably, CD4+CD25−FoxP3+ population is also increased in systemic lupus erythematosus patients [66], but its nature remains undetermined. Some authors suggested that these cells are FoxP3+ non-Treg T cells [67], whereas others argue that these cells are dysfunctional Treg cells with limited regulatory potential [68].

In order to clarify this, we evaluated FoxP3 liability in these cells by keeping them unstimulated for different periods of time. By doing this, we observed that within these cells Foxp3 expression was relatively stable, suggesting that, at least in HIV-TB patients, the intriguing CD4+CD25−FoxP3+ population is not a transiently activated effector population and rather might have regulatory functions [67, 68]. We hypothesize that this unusual regulatory population may preclude protective immune responses against* Mtb* in our cohort of coinfected patients, a topic currently under investigation by our group. Finally, we also found that the frequency of CD4+CD25−FoxP3+ cells in HIV-TB patients negatively correlated with DHEA plasma levels, which is consistent with a role of DHEA on enhancing Th1 responses while diminishing this particular regulatory T cell population.

Moreover, we also found that the initiation of antituberculous treatment (ATT) diminished CD4+CD25−FoxP3+, which was restored to normal levels after finalization of treatment. In contrast, conventional CD4+CD25+FoxP3+ Tregs in HIV-TB patients tended to diminish across visits, but not significantly. In HIV-TB patients, adrenal hormone balance was not restored as well, at least after 6 months of ATT (unpublished results).

In some HIV-infected patients, the recovery of specific immune responses during the beginning of highly active antiretroviral treatment (HAART) can elicit systemic inflammatory responses which lead to the establishment of the immune reconstitution inflammatory syndrome (IRIS). In HIV-TB IRIS patients, DHEA-s plasma levels were three times lower and cortisol/DHEA ratio was up to four times higher compared to the non-TB groups. The CD4+CD25−FoxP3+ frequency was also increased in IRIS patients and negatively correlated with DHEA-s plasma levels [65].

Previous results obtained by our research group also showed a positive correlation between DHEA plasma levels and the frequency of a terminally differentiated population of CD8+ T cells in HIV-TB patients, which is thought to be crucial in preventing TB reactivation (in press and [38]). We also observed that* in vitro* DHEA treatment increased* Mtb*-specific CD8+ T cell proportions and terminal differentiation in CD8+ T cells of HIV-TB coinfected patients. Additionally, we found that DHEA* in vitro* increased the expression of the transcription factor Tbet and Tbet/Eomesodermin ratio in isolated CD8+ cells, both known to drive to terminal differentiation in CD8+ T cells [69].

Based on our results, in HIV-TB coinfected patients, HPA axis displays a noticeable alteration, probably influenced by the chronic state of inflammation induced by both pathogens concomitantly. This condition may contribute to the immune system dysfunction seen in these patients.

## 5. Adrenal Steroids in Immunotherapy

As suggested by the evidence depicted above, DHEA seems to have two different functions regarding immune modulation: it favors Th1 responses, fundamental in the protection against intracellular pathogens like* Mtb* and HIV, and, on the other hand, it exerts anti-inflammatory actions, which may be beneficial in the context of chronic infection. For that reason, DHEA has been proved as an adjuvant therapy for the treatment of several infectious diseases.

The apparent dual function of DHEA, together with the fact that a cell receptor is not known for it, suggests that this hormone may exert its immune regulatory function through some of its metabolites, which are known to be potent immune-regulators* in vivo* [26]. DHEA is the physiological precursor for the synthesis of androgens and estrogen, but it can also be metabolized into oxygenated derivatives in nonsteroidogenic tissues, especially in human liver [70]. There is also evidence that DHEA can be converted into oxygenated derivatives like 3*β*,17*β*-androstenediol (AED) and 3*β*,16*β*,17*β*-androstenetriol (AET) by monocyte-derived macrophages [71].

In mice, DHEA and AED were protective against lethal dose of* Pseudomonas aeruginosa* or* Enterococcus faecalis* infection. Both steroids appeared to act on immune system rather than bacteria directly, since the growth of bacterium was not altered in* in vitro* assays [72]. DHEA, AET, and AED also proved to protect mice against lethal doses of human herpes type 2 or coxsackievirus B4 [26, 72] while DHEA supplementation enhanced the immune response against* Trypanosoma cruzi* in mice, as shown by a significant reduction in parasitemia levels [73].

A synthetic derivate from DHEA, 16*α*-Bromoepiandrosterone (HE2000), has been tested in animal models of TB [25]. In these sets of experiments, HE2000 proved to reduce bacterial load associated with progressive TB and lowered IL-4 expression associated with* Mtb* infection. Also, BALB/c mice with active TB treated with HE2000 showed lower bacterial proliferation and a higher Th1/Th2 cytokine balance. The treatment with HE2000 also resulted in lower percentages of lungs involved in pneumonia and higher bacterial clearance, when administered as an adjunct to conventional chemotherapy [25].

The above mentioned evidence suggests that DHEA and its derivate upregulate host immunity, leading to higher resistance to infectious agents. In line with this, treatment of mice with AET augmented the absolute numbers of CD4+/CD8+ T cells after irradiation and increased IL-2, IL-3, and IFN-*γ* levels counteracting hydrocortisone immune suppression [26]. Additionally, zinc and DHEA supplementation augmented macrophage count and IFN-*γ* and NO concentrations in an additive manner [24].

Despite the above mentioned immune-enhancing effects of DHEA, the effect of this steroid on T cell function is controversial. While some researchers showed that DHEA enhanced T cells proliferation and IL-2 production [70], others reported the contrary [74]. A posterior study suggested that DHEA acted differentially on T and B lymphocytes, since it significantly inhibited proliferation of T cells, but, on the other hand, increased B cell mitogen pokeweed mitogen (PWM) effect on PBMC [75].

Experiments performed by our group demonstrated that DHEA induced the increment in the production of IL-12 and reduced IL-10 secretion by* Mtb*-stimulated human monocyte-derived dendritic cells (DCs) promoting Th1 responses [76]. Also, DHEA enhanced the expression of MHC I, MHC II, and CD86 and increased ERK1/2 phosphorylation. Furthermore, DHEA enhanced the antigen-specific T cell proliferation and IFN-*γ* production induced by* Mtb*-stimulated DCs.

DHEA synthetic derivate, HE2000, also proved to be beneficial in human infection. A pilot study showed a 50% reduction in* Plasmodium falciparum* blood levels in 41 out of 42 patients enrolled, thereafter achieving undetectable parasitemia levels in 76% of them [77]. A randomized, double-blind, placebo-controlled study showed that HE2000 administration induced significantly sustained decreases in IL-1*β*, TNF-*α*, IL-6, and Cox-2 transcripts and although CD4+ T cell numbers remain unaltered, patients demonstrated a significant decrease in viral load and a significant increase in CD8+ T cell responses after HE2000 administration [27]. Noteworthy, as shown by another study, HE2000 lowered by 42.2% the incidence of tuberculosis coinfection in AIDS patients while also reduced the cumulative incidence of opportunistic infections [78]. Unfortunately, despite the positive results obtained in phase I and II studies, currently there are no ongoing clinical trials exploring the effects of HE2000. Finally, at present we are studying the modulatory features of 7-oxo-DHEA, a DHEA natural derivative, on the immune response against TB in the context of HIV infection.

## 6. Concluding Remarks

Immune endocrine interactions during infectious diseases may determine the failure or success of the immune response. This is particularly true for chronic infections like HIV or TB, in which pathogens and immune system coexist in a long struggle. Here we show the importance of DHEA and its derivatives on immunity to intracellular pathogens like HIV and* Mtb* through its action on Th1 and CD8 responses. These compounds also exert anti-inflammatory actions, which may be beneficial in the context of chronic infections, especially during HIV-TB coinfection.

This dual function of DHEA makes it very suitable as an immune-therapy for the prevention and treatment of TB in the context of HIV infection.
